# Supporting local diversity of habitats and species on farmland: a comparison of three wildlife‐friendly schemes

**DOI:** 10.1111/1365-2664.12557

**Published:** 2015-11-18

**Authors:** Chloe J. Hardman, Dominic P.G. Harrison, Pete J. Shaw, Tim D. Nevard, Brin Hughes, Simon G. Potts, Ken Norris

**Affiliations:** ^1^ Centre for Agri‐Environmental Research School of Agriculture, Policy and Development University of Reading Reading RG6 6AR UK; ^2^ Engineering and the Environment University of Southampton Highfield Southampton SO17 1BJ UK; ^3^ Research Institute for the Environment and Livelihoods Charles Darwin University Darwin NT0909 Australia; ^4^ Conservation Grade 2 Gransden Park Abbotsley Cambridgeshire PE19 6TY UK; ^5^ Institute of Zoology Zoological Society of London London NW1 4RY UK

**Keywords:** agri‐environment schemes, bees, birds, butterflies, landscape heterogeneity, organic farming, plants, pollinators

## Abstract

1. Restoration and maintenance of habitat diversity have been suggested as conservation priorities in farmed landscapes, but how this should be achieved and at what scale are unclear. This study makes a novel comparison of the effectiveness of three wildlife‐friendly farming schemes for supporting local habitat diversity and species richness on 12 farms in England.
2. The schemes were: (i) Conservation Grade (Conservation Grade: a prescriptive, non‐organic, biodiversity‐focused scheme), (ii) organic agriculture and (iii) a baseline of Entry Level Stewardship (Entry Level Stewardship: a flexible widespread government scheme).
3. Conservation Grade farms supported a quarter higher habitat diversity at the 100‐m radius scale compared to Entry Level Stewardship farms. Conservation Grade and organic farms both supported a fifth higher habitat diversity at the 250‐m radius scale compared to Entry Level Stewardship farms. Habitat diversity at the 100‐m and 250‐m scales significantly predicted species richness of butterflies and plants. Habitat diversity at the 100‐m scale also significantly predicted species richness of birds in winter and solitary bees. There were no significant relationships between habitat diversity and species richness for bumblebees or birds in summer.
4. Butterfly species richness was significantly higher on organic farms (50% higher) and marginally higher on Conservation Grade farms (20% higher), compared with farms in Entry Level Stewardship. Organic farms supported significantly more plant species than Entry Level Stewardship farms (70% higher) but Conservation Grade farms did not (10% higher). There were no significant differences between the three schemes for species richness of bumblebees, solitary bees or birds.
5. *Policy implications*. The wildlife‐friendly farming schemes which included compulsory changes in management, Conservation Grade and organic, were more effective at increasing local habitat diversity and species richness compared with the less prescriptive Entry Level Stewardship scheme. We recommend that wildlife‐friendly farming schemes should aim to enhance and maintain high local habitat diversity, through mechanisms such as option packages, where farmers are required to deliver a combination of several habitats.

## Abstract 2

The wildlife‐friendly farming schemes which included compulsory changes in management, Conservation Grade and organic, were more effective at increasing local habitat diversity and species richness compared with the less prescriptive Entry Level Stewardship scheme. We recommend that wildlife‐friendly farming schemes should aim to enhance and maintain high local habitat diversity, through mechanisms such as option packages, where farmers are required to deliver a combination of several habitats.

## Introduction

The expansion and intensification of agricultural land is a global threat to biodiversity (Green *et al*. 2005), and biodiversity declines associated with agricultural intensification have been documented for multiple taxa (birds: Donald *et al*. 2006; aculeate pollinators: Ollerton *et al*. 2014; Lepidoptera: Ekroos, Heliölä & Kuussaari 2010; plants: Kleijn *et al*. 2009). Agricultural intensification reduces the spatial and temporal complexity of habitats (Stoate *et al*. 2001). This reduction in habitat heterogeneity has occurred at multiple spatial scales, for example through reduced crop diversity and hedgerow removal at local scales and homogenization of land‐use types at landscape scales (Tscharntke *et al*. 2005). Restoring habitat heterogeneity has been proposed as a ‘universal management objective’ that would increase biodiversity in agricultural systems (Benton, Vickery & Wilson 2003). However, the suitability of this objective has been disputed for low‐intensity agricultural landscapes (Batáry *et al*. 2011a). In agricultural landscapes, relationships between habitat diversity and species richness are taxon specific and scale dependent (Jeanneret, Schüpbach & Luka 2003; Weibull, Ostman & Granqvist 2003; Gaba *et al*. 2010). Therefore, how habitat diversity should be restored and at what scale are questions that need further research.

In Europe, government‐run agri‐environment schemes (AES) and private sector environmental certification schemes are important mechanisms for reducing the negative environmental impacts of agricultural intensification. Government AES encompass a range of financial incentives for farmers to undertake low‐input extensive farming and/or restoration of particular habitats, species or landscape features (Hart 2010). The effectiveness of AES in conserving and promoting biodiversity has been highly variable; depending on ecological contrast, landscape context and land‐use intensity (Kleijn *et al*. 2011). AES appear to be most effective when they create a high ecological contrast (the extent to which the AES management improves habitat conditions for the target group relative to conventional management, Scheper *et al*. 2013). In addition, there is evidence that AES are most effective in simple landscapes (1–20% semi‐natural habitat), compared to complex (>20%) (Batáry *et al*. 2011b).

Environmental Stewardship is an English AES, with a wildlife conservation focus, which accepted applications between 2005 and 2013 (Natural England 2013a). The scheme has two tiers of whole‐farm schemes: Entry Level Stewardship (ELS, 5‐year agreements) and Higher Level Stewardship (HLS, 10‐year agreements in addition to ELS). ELS is a ‘broad and shallow’ scheme, which aimed to maximize geographic coverage. ELS includes management options for boundary features, trees and woodland, historic and landscape features, buffer strips, arable, grassland, crop diversity and soil and water protection. Each option gains a number of points per unit area, and farmers choose how to combine options to achieve an overall 30 points per hectare. The organic version of ELS (OELS) includes the same choice of options, and farmers are paid double the conventional rate. In contrast, HLS is a ‘narrow and deep’ scheme, which is regionally targeted and competitive. HLS contains more complex management options including the creation, restoration and maintenance of priority habitats, such as species‐rich semi‐natural grassland. ELS covered 64·6% of England's agricultural land area in October 2013, OELS covered 3·4%, and HLS covered 13·0% (Natural England 2013b).

Direct comparisons of organic farms with non‐organic biodiversity‐targeted AES are scarce (but see Marja *et al*. 2014). This research gap was highlighted by Hole *et al*. (2005). Studies examining the different AES in England have shown effectiveness to be variable. Organic farming has been evaluated extensively, and a recent meta‐analysis showed that it was associated with 30% greater species richness compared to conventional farming (Tuck *et al*. 2014). Benefits of HLS have been observed for birds (Bright *et al*. 2015), whilst ELS has been shown to benefit granivorous passerines in winter, but to have mixed effects during the breeding season (spring/summer, Baker *et al*. 2012). The impacts of ELS on birds and pollinators have been limited by low uptake of the most effective options (Butler, Vickery & Norris 2007; Breeze *et al*. 2014). At a national scale, hedgerow management and low‐input grassland together account for half of all points awarded in ELS (Breeze *et al*. 2014). Farmers did not need to change existing management in 50% of cases for hedgerow options and 81% of cases for low‐input grassland options in order to qualify for ELS payments (Boatman *et al*. 2007).

In addition to government AES, farmers can enter ecological certification schemes. One such scheme, which has more stringent habitat management requirements than ELS, is Conservation Grade (CG, http://www.conservationgrade.org). This scheme uses a ‘Fair to Nature’ protocol that requires 10% of the farm area to be managed solely for wildlife habitat according to a specific formula: 4% pollen‐ and nectar‐rich habitats, including a grass and native wildflower mix (>1·5%) and a legume mix (<2·5%); 2% wild bird food crops, including at least three seed‐producing crops such as barley, triticale, kale or quinoa; 2% tussocky and fine grasses; and 2% wildlife habitat specific to the farm. Pollen and nectar habitats and wild bird food crops require continued management to maintain quality. The additional management costs are met through sales of ‘Fair to Nature’ branded food products. CG has been implemented since 2004 and currently involves 80 farms, mostly cereal producers in the UK. CG farms had on average 24 times more nectar flower mixture (EF4) and 15 times more wild bird seed mixture (EF2) than farms in ELS alone (Natural England 2013a, proportional area data from 52 CG farms).

The CG protocol was based on evidence from experimental farms that showed significantly higher levels of invertebrates in sown margin mixes compared to the crop (Meek *et al*. 2002) and substantial benefits of pollen and nectar mixes for bumblebees (Carvell *et al*. 2004). More recently, benefits of sown wildflower strips for insects have been demonstrated more widely (Haaland, Naisbit & Bersier 2011) and wild bird food crops have been found to support higher densities of birds in winter compared to controls (Henderson, Vickery & Carter 2004; Hammers *et al*. 2015). Taxon‐specific studies have been carried out in parallel with our multitaxa study, using a subset of the same sites. CG farms supported higher densities of granivorous passerines in winter than organic farms (Harrison 2013), and functional diversity of hoverflies on CG farms was slightly higher and less variable between farms (Cullum 2014) compared to organic. We undertook the first multitaxa study of farmer‐managed CG farms and examined how they compared to alternative wildlife‐friendly farming schemes.

We compared CG, organic and ELS, in terms of the extent to which they enhanced habitat diversity and species richness of a wide range of taxa. We focused on spatial rather than temporal heterogeneity and on habitat diversity rather than configuration. We examined habitat diversity at multiple spatial scales, since not doing so would potentially miss important species–landscape effects (Jackson & Fahrig 2015). This analysis also enabled us to check whether scheme type was associated with landscape diversity.

Our research questions were: (i) Does habitat diversity vary between these wildlife‐friendly farming schemes at local and landscape scales? (ii) At which spatial scale does species richness of different taxonomic groups respond to habitat diversity? (iii) Does species richness differ between farms in the three schemes and if so, how far can this be explained by habitat diversity? We collected spatial data on habitats, along with species richness and abundance data on plants, butterflies, bumblebees, solitary bees and birds in order to answer these questions. We expected farms in the additional schemes (CG and organic) to support higher species richness and habitat diversity than farms only in ELS. We expected taxonomic groups to respond most strongly to habitat diversity at scales similar to those at which individuals typically use the landscape. We expected local habitat diversity to be more important on CG and ELS farms than on organic farms, since organic crops receive lower or zero synthetic chemical inputs compared to CG and ELS. Therefore, an organic area surrounded by low habitat diversity would be expected to support more species than a non‐organic equivalent.

## Materials and methods

### Defining spatial scales

We evaluated habitat diversity at four spatial scales: two local scales that largely reflect within‐farm management (100‐m radius; 3·14 ha and 250‐m radius; 19·6 ha) and two larger scales that represent the wider landscape (1‐km radius; 314 ha and 3‐km radius; 2827 ha). These radii were chosen because they cover the range of radii at which different taxonomic groups have been found to typically use the landscape: birds, up to 3 km (Pickett & Siriwardena 2011); bumblebees, up to 2 km (Walther‐Hellwig & Frankl 2000); solitary bees, up to 600 m (Gathmann & Tscharntke 2002); and butterflies, up to 420 m (Merckx & Van Dyck 2002).

### Study sites

**Figure 1:**
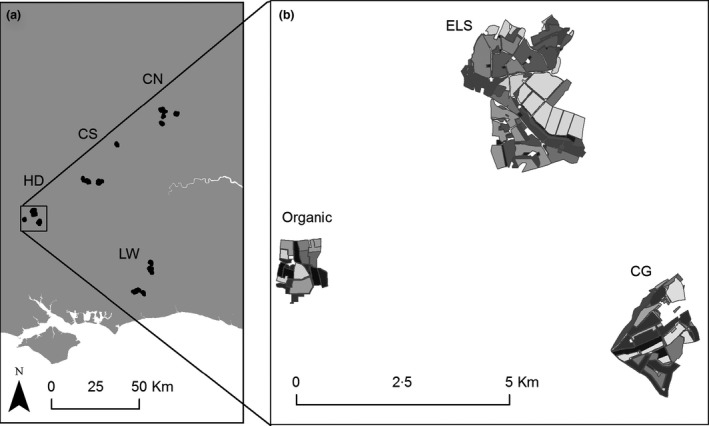
Sampling maps showing (a) the location of the four regions in southern England: HD, Hampshire Downs; CS, Chilterns South; CN, Chilterns North; LW, Low Weald and (b) one region containing a triplet of farms one in each wildlife‐friendly farming scheme: ELS, Entry Level Stewardship; CG, Conservation Grade; and organic.

This study was carried out in southern England on matched triplets of farms to minimize confounding environmental variables. Triplets of sites were matched on region (Joint Character Areas [Natural England 2011]), soil type (NSRI 2011), crops and livestock, as far as possible. The number of sites fitting these selection criteria was low, but four suitable triplets were found (Fig. 1, Table S1, Supporting Information). There were no significant differences in broad habitat composition metrics between scheme types (farm scale and 1‐km radius scale, Tables S2 and S3). The minimum time since scheme entry was 6 years for CG farms and 5 years for ELS farms (with one exception of 2 years). The minimum time since organic conversion started was 13 years. Three‐quarters of the CG and organic farms were in HLS, and one organic farm began HLS conversion towards the end of the study. Nationally, 56% of CG, 25% of OELS farms and 24·5% of ELS farms were in HLS in 2013 (Natural England 2013a).

Average farm size was 267·5 ± 36·6 ha (mean ± SE), and the average field size was 9·11 ± 0·40 ha. Organic farms had significantly smaller field sizes than the ELS farms (chi‐square test [2] = 5·43, *P *=* *0·021) and significantly lower wheat yields than ELS farms (generalized linear mixed models [GLMM] chi‐square test [2] = 13·70, post hoc tests: CG > Org: *P *=* *0·001, ELS > Org: *P *=* *0·005) (Table S4). CG farms had a higher number of HLS options per farm than organic farms (chi‐square test [2] = 16·148, *P *=* *0·001). However, there were no differences between schemes in the number of ELS options per farm (chi‐square test [2] = 7·319, *P *=* *0·292).

### Habitat mapping

Farm habitats were mapped by digitizing Environmental Stewardship maps and cropping plans using Arc GIS v.10, with a minimum mappable unit of 0·01 ha. The UK Land Cover Map 2007 was used as a base for landscape mapping (Centre for Ecology & Hydrology 2011), which has a minimum mappable unit of 0·5 ha. All maps were ground‐truthed (Methods detailed in Appendix S1, habitat categories in Tables S5 and S6).

### Biodiversity sampling strategy

A proportional stratified sampling technique was designed to represent the habitat composition of each farm. If calculated by area alone, Environmental Stewardship options of high biodiversity value covering small areas would be under‐represented; therefore, areas of AES options were weighted using the points scored in ELS/OELS/HLS (for details see Appendix S1). Sampling stations were plotted randomly according to habitat designations using the ‘genrandompnts’ tool (Beyer 2012).

### Habitat diversity calculations

Habitat diversity was calculated using a Shannon diversity index, which emphasizes rare habitat types that may be important for sensitive species (Nagendra 2002). To avoid bias in the comparison of habitat diversity between schemes, landscape buffers were drawn around random points. The same number of points was generated as the number of sampling points used for biodiversity surveys. To test correlations between species richness and habitat diversity, buffers were generated around biodiversity sampling stations and clipped to relevant habitat maps.

### Biodiversity survey methods

Biodiversity surveys were carried out between 2012 and 2014, between April and August. An additional winter bird survey was carried out between January and March 2013, but only in three of the four regions due to logistical constraints. Sampling effort varied between years, but was always consistent within years, with five sampling rounds for summer birds, three for insects and winter birds and one for plants, at 10–30 sampling points per farm. Butterflies were recorded on transects using UK butterfly monitoring methods (Pollard & Yates 1993); bees were sampled using triplicate pan traps (Westphal *et al*. 2008) and identified to species using keys (solitary bees: Else In Press; bumblebees: Prŷs‐Jones & Corbet 2011). Birds were sampled along line transects using similar methods to the British Breeding Bird Survey, and plants were surveyed in 1‐m^2^ quadrats at each pan trap sampling point (further method details in Appendix S1).

### Statistical analysis

We accounted for the nested design by including farm nested in region as a random effect. All GLMM were fitted using the package lme4 (Bates *et al*. 2014). Models were checked for overdispersion and residual normality and heteroscedascity. Conditional and marginal *R* ^2^ were calculated (Nakagawa & Schielzeth 2013). Likelihood ratio tests (LRTs) were used to assess the significance of terms in the models (Zuur *et al*. 2009). Post hoc simultaneous tests for general linear hypotheses using single‐step *P* value adjustments were made to correct for multiple comparisons (multcomp package, Hothorn, Bretz & Westfall 2008). All analyses were performed using R v. 3.1.1 (R Core Team 2014).

#### Wildlife‐friendly farming scheme differences in habitat diversity

To test the effect of scheme type and buffer radius on habitat diversity, we used a GLMM estimated using ML with Gaussian errors. Buffer radius length was categorical, and the interaction between radius and scheme type was examined. Year was a random effect since it represented temporal autocorrelation and did not influence the mean habitat diversity (GLMM LRT for year as a fixed effect, chi‐square test [1] = 2·699, *P *=* *0·100). The nested random effects structure was the following: Year/Region/Farm/Point since the data included multiple buffers around the same points.

#### Habitat diversity as a predictor of species richness

Species richness data were pooled across sampling rounds. Habitat diversity at each spatial scale was tested as a predictor of species richness of different taxonomic groups in separate GLMM models. Bonferroni corrections were not used, in order to retain statistical power (Nakagawa 2004). Year was a fixed effect since species richness varied significantly between years. For summer bird models, where there were several observers, observer was included as a random effect. For birds and insects, abundance was included as a fixed effect to account for sample size variation. The potentially confounding influence of 1‐km landscape proportion of mass flowering crop was included in insect models. Proportion of semi‐natural habitat in the surrounding landscape was not included because it was significantly correlated with habitat diversity at landscape scales (GLMM 3 km: estimate: 0·010 ± 0·002, LRT chi‐square test = 29·359, *P *<* *0·001; 1 km: estimate: 0·005 ± 0·001, LRT chi‐square test = 8·405, *P *=* *0·004). For butterflies, birds and bumblebees, a Poisson distribution was used. For solitary bees and plants, the log‐normal Poisson (Elston *et al*. 2001) and negative binomial distributions were used, respectively, to reduce overdispersion.

#### Effects of wildlife‐friendly farming scheme and habitat diversity on species richness

To test for the effect of scheme type on species richness, we used GLMM models that included fixed effects for year. The proportion of mass flowering crop in a 1‐km radius buffer was included for models on insects. Subsequently, we tested for interactions between scheme type and habitat diversity at the 100 and 250‐m scales and then carried out model simplification according to the guidance of Zuur *et al*. (2009). We did not explore interactions between landscape habitat diversity and scheme type because there was not sufficient replication at the landscape scale to draw valid conclusions. By putting habitat diversity and scheme type into models together, we could evaluate the relative effects of each variable on species richness.

## Results

### Wildlife‐friendly farming scheme differences in habitat diversity

**Figure 2:**
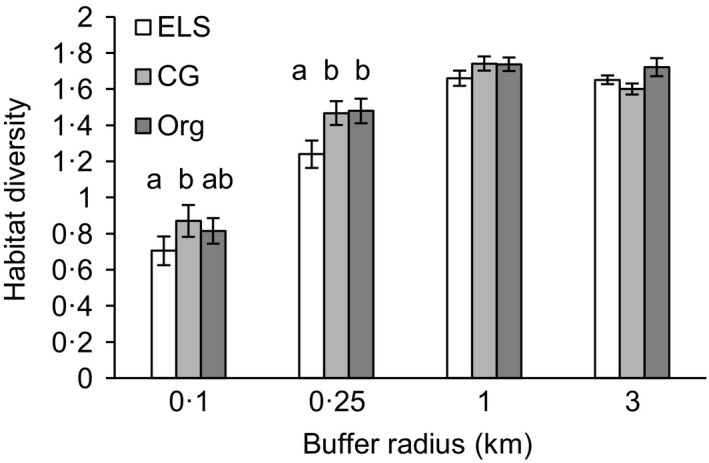
Variation in Shannon habitat diversity at different spatial scales for farms in three different wildlife‐friendly farming schemes: ELS, Entry Level Stewardship; CG, Conservation Grade; Org, organic. Means and 95% confidence intervals from the raw data are shown. Letters a and b indicate significant differences between schemes at *P* < 0.05.

Differences in habitat diversity between scheme types varied with spatial scale, with significant differences at local but not at landscape scales (GLMM scheme type × radius interaction LRT: chi‐square test [6] = 38·64, *P *<* *0·001, Fig. 2, Table S7). CG farms supported 24% higher habitat diversity than ELS at the 100‐m scale and 18% higher habitat diversity at the 250‐m scale (post hoc tests *P *=* *0·021 and *P *<* *0·001, respectively). Organic farms supported 16% higher habitat diversity at the 100‐m scale (*P *=* *0·109) and 19% higher habitat diversity than ELS at the 250‐m scale (*P *<* *0·001).

### Habitat diversity as a predictor of species richness

**Figure 3:**
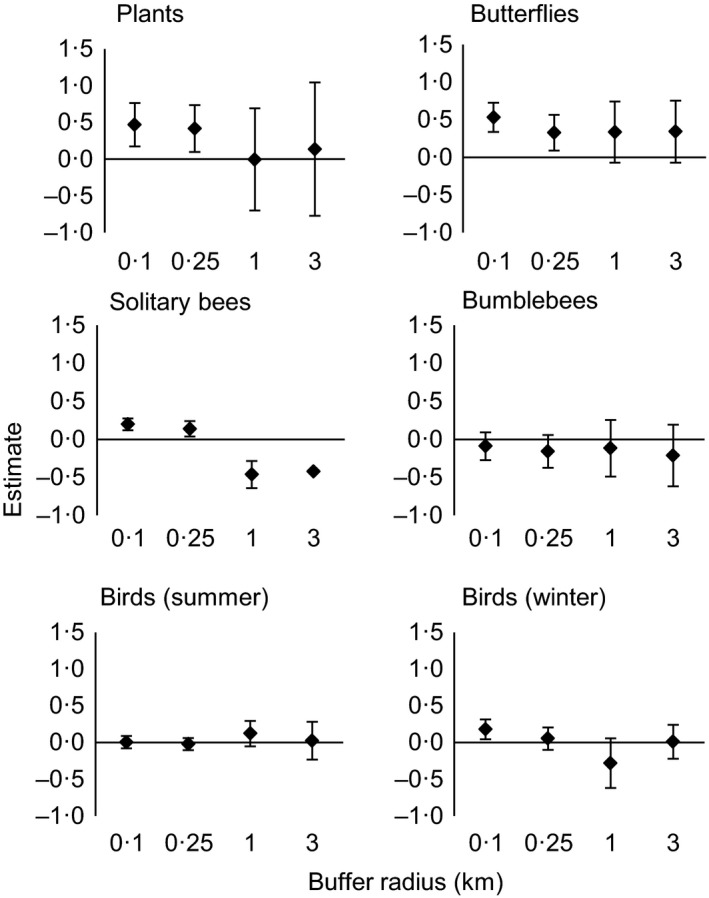
Effect sizes (and 95% confidence intervals) from models using habitat diversity to predict species richness, repeated for four spatial scales and six taxonomic groups.

During this study, we recorded the following numbers of species: 23 butterflies; 84 solitary bees; 14 bumblebees; 95 birds in summer; 59 birds in winter; and 178 plants (of which 123 were insect‐rewarding; M. Baude, pers. comm.). Relationships between species richness and habitat diversity varied between taxonomic groups (Fig. 3, Table S8). For butterflies, solitary bees, plants and winter birds, habitat diversity at the 100‐m radius scale significantly predicted species richness (butterflies: *P *<* *0·001, plants: *P *<* *0·001, solitary bees: *P *=* *0·014, winter birds: *P *=* *0·012). Significant positive effects of habitat diversity at the 250‐m scale were seen for butterflies (*P *=* *0·006) and plants (*P *=* *0·012). There was a negative effect of habitat diversity at the 1‐km scale for species richness of solitary bees (*P *=* *0·029). For summer birds and bumblebees, no significant correlations between species richness and habitat diversity were seen at any spatial scale.

### Effects of wildlife‐friendly farming scheme and habitat diversity on species richness

**Figure 4:**
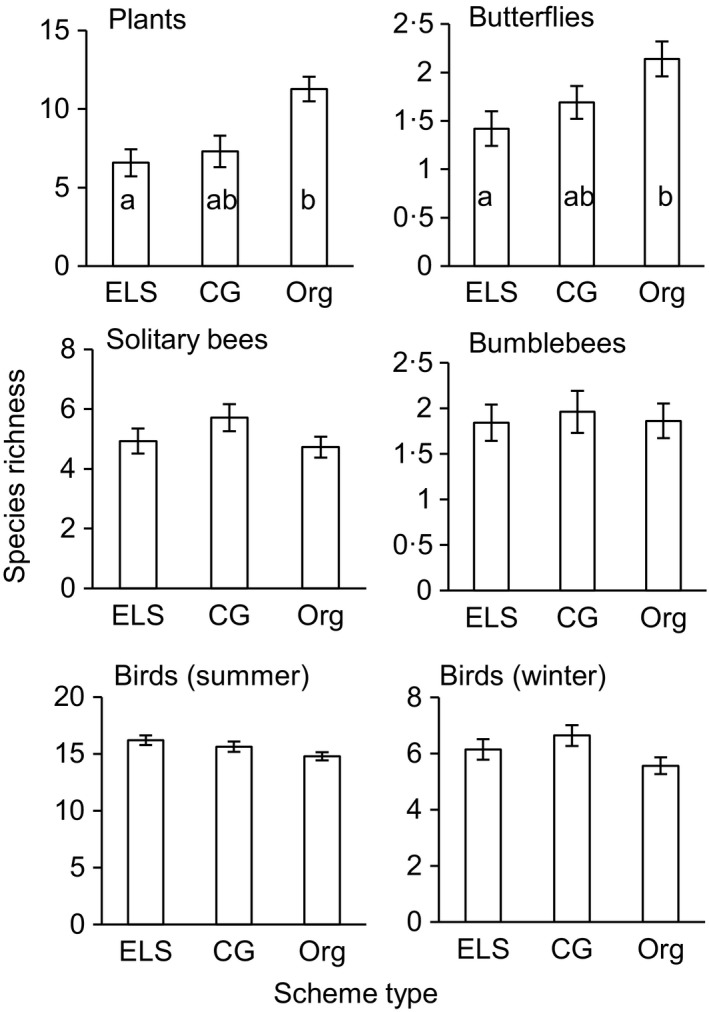
Variation in species richness per sampling point pooled across years for farms in three different wildlife‐friendly farming schemes: ELS, Entry Level Stewardship; CG, Conservation Grade; Org, organic. Means and 95% confidence intervals from the raw data are plotted with y‐axes scaled appropriately for each taxonomic group. Letters a and b indicate significant differences between schemes at *P* < 0.05.

The schemes had varying effects on species richness per sampling point, depending on taxonomic group (Fig. 4, Table 1). Butterfly species richness was 50% higher on organic farms compared to ELS farms (*P *=* *0·046) and 20% higher on CG farms compared to ELS farms (*P *=* *0·062). Plant species richness on organic farms was 70% higher compared to ELS farms (*P *=* *0·013) and 60% higher compared to CG farms (*P *=* *0·067). No other significant differences between scheme types were seen. Species richness at the farm scale did not vary between scheme types (Friedman chi‐square tests: plants, chi‐square test [2] = 0·5, *P *=* *0·789; butterflies, chi‐square test [2] = 2·6, *P *=* *0·273; bumblebees, chi‐square test [2] = 2·923, *P *=* *0·232; solitary bees, chi‐square test [2] = 0·5, *P *=* *0·789; summer birds, chi‐square test [2] = 2, *P *=* *0·368; winter birds: chi‐square test [2] = 2, *P *=* *0·368).

**Table 1:** Results of generalized linear mixed models testing for differences in species richness between wildlife‐friendly farming schemes, with significant differences at *P* < 0.05 shown in bold

Scheme‐type likelihood ratio test			Post hoc test		Marginal *R* ^2^	Conditional *R* ^2^
	Chi‐square test (2 df)	*P* value	Direction	*P* value		
Plants	**6·678**	**0·035**	**Org > ELS**	**0·013**	0·537	0·552
			Org > CG	(0·067)		
Butterflies	**7·093**	**0·029**	**Org > ELS**	**0·046**	0·936	0·936
			CG > ELS	(0·062)		
Bumblebees	1·577	0·454			0·686	0·686
Solitary bees	1·202	0·548			0·415	0·680
Birds (summer)	1·118	0·572			0·945	0·949
Birds (winter)	1·220	0·543			0·409	0·417

CG, Conservation Grade; ELS, Entry Level Stewardship; Org, organic.

No interactions between local habitat diversity and scheme type were significant in explaining species richness. Testing scheme type and local habitat diversity as predictors of species richness together produced largely the same results as testing independently. The only difference was for butterflies where, once habitat diversity at the 250‐m scale was included in models, the effect of scheme type was no longer significant (LRT chi‐square test = 5·26, *P *=* *0·072, Table S9).

## Discussion

The results showed that farms in additional wildlife‐friendly farming schemes (CG and organic) supported higher habitat diversity than farms in the ‘broad and shallow’ ELS scheme. The higher local habitat diversity on CG farms was likely to be due to the greater number of HLS options per farm. Organic agriculture per se does not prescribe non‐crop habitat management, but the higher habitat diversity on organic farms could be due to the significantly smaller fields (an organic attribute also found more widely, Norton *et al*. 2009) and/or the HLS scheme. The farms in our study met the minimum requirements for the schemes we were interested in (CG, ELS and organic). However, farmers can carry out additional wildlife‐friendly management beyond the minimum requirements set by these schemes. Three‐quarters of the farms in CG and organic schemes carried out additional management as part of the HLS scheme. In interpreting the results, we need to be aware that the differences seen in the CG vs. ELS and organic vs. ELS comparisons may have been amplified by the HLS scheme. Further research with a larger sample size of farms could investigate the individual and aggregate impacts of combined schemes.

We found stronger associations between sampling point species richness and local habitat diversity (100 or 250‐m radius) compared to landscape (1 or 3‐km radius) habitat diversity. These effects depend upon the degree to which land‐use classifications reflect suitable habitats for species in the area. Had higher resolution habitat maps for the landscape scale been available, positive effects of landscape habitat diversity on species richness may have been apparent; land‐use maps of relatively larger grain were employed in the present study.

Positive correlations between species richness and local habitat diversity were seen for plants, butterflies and solitary bees. This conformed to our expectation that animal taxa with smaller home ranges would respond more strongly to local scale habitat diversity at local scales (100 and 250‐m radii). Positive effects of habitat heterogeneity on species diversity have been found for plants at the 200‐m scale in cereal fields in France (Gaba *et al*. 2010), and for butterflies at the 500‐m scale in the UK (Botham *et al*. 2015). Points with high habitat diversity at the 100‐m‐radius scale are often near field edges or in non‐crop habitats. Field edges are commonly found to support more species than field centres (e.g. Gabriel *et al*. 2010). Field edges are likely to have higher plant species richness since they tend to have lower agrochemical exposure and may receive plant propagules from neighbouring habitats (Zonneveld 1995). In addition, bird species richness in winter showed a positive correlation with local habitat diversity, but bird species richness in summer did not. This could be because AES management for winter food resources has a stronger effect than management for breeding season resources (as found by Baker *et al*. 2012). In our study, there could also be a sampling effect, since all summer bird transects were along boundaries due to access limitations, whereas winter bird sampling points also sampled field centres so included more points with low habitat diversity.

The results suggest that landscape moderation of AES effectiveness was occurring, since a negative relationship between solitary bee species richness and landscape habitat diversity at the 1‐km scale was found. This fits with the intermediate landscape‐complexity hypothesis, proposed by Tscharntke *et al*. (2005) and supported by evidence (Batáry *et al*. 2011a), in which AES in simple landscapes are more effective. If we had sampled more triplets of farms in simple landscapes, we expect to have seen more significant benefits of the CG scheme. Based on these results and the wider literature (Carvell *et al*. 2011; Scheper *et al*. 2013; Wood, Holland & Goulson 2015), we recommend that the CG scheme targets low‐diversity landscapes.

The benefits of CG and organic farming for species richness varied between taxa. No effects were seen for bumblebees or summer birds. This is perhaps because bumblebees and birds use the landscape at larger scales than individual farms. Perhaps if the CG or organic schemes were implemented throughout a landscape, positive effects for bumblebees and birds would be found. The limited benefit of organic farming for birds is consistent with Chamberlain, Wilson & Fuller (1999) and Gabriel *et al*. (2010) but in contrast to the findings of Hole *et al*. (2005) and Bengtsson, Ahnström & Weibull (2005), showing how variable the impact of organic farming can be on birds.

Differences between scheme types in butterfly species richness were no longer significant once habitat diversity at the 250‐m radius scale was included in models. This suggests that the effect of the organic and CG schemes on butterfly species richness was partly mediated through the effect of habitat diversity. For plants, organic farming remained beneficial even once habitat diversity was taken into account. This was expected due to plant species richness commonly benefitting from organic farming (Tuck *et al*. 2014) due to reduced agrochemical use (Geiger *et al*. 2010).

The three schemes examined are all examples of land‐sharing (Phalan *et al*. 2011). However land‐sparing offers an opportunity to protect or restore natural habitat and associated species by intensifying yields on existing land to prevent further agricultural land conversion (Phalan, Green & Balmford 2014). There is potential to intensify yields using ecosystem services rather than synthetic inputs (Bommarco, Kleijn & Potts 2013), and the capacity of each scheme in achieving this could be investigated in future. A wider analysis of trade‐offs between yields and biodiversity for each of these schemes could also be explored. It is worth noting that in this study, CG farms outperformed organic farms on wheat yields by up to 5 tonnes per hectare whilst still supporting 20% more butterfly species than ELS farms.

### Conclusions and policy recommendations

Our study confirms that increasing local habitat diversity is a valid objective in high‐intensity agricultural landscapes, since it is associated with biodiversity benefits. There will be a threshold past which further increase in habitat heterogeneity will be detrimental due to shrinking patch size reducing viable populations (Fahrig *et al*. 2011; Redon *et al*. 2014), and the threshold for this effect in AES systems needs further research. Three broad (but not mutually exclusive) mechanisms by which local habitat diversity can be increased are by: (i) increasing non‐crop habitat diversity (typical of CG, ELS and HLS schemes), (ii) increasing crop diversity (Le Féon *et al*. 2013) and (iii) reducing the grain of the landscape by reducing field size (Fahrig *et al*. 2015) through restoring hedgerows and field margins.

Recent policy changes that are likely to influence local habitat diversity have occurred in the EU. The Common Agricultural Policy reform 2014–2020 made 30% of the ‘Pillar 1’ direct payments to farmers dependent on three compulsory greening rules: protection of permanent grassland, diversification of crop measures and maintenance of ecological focus areas. Although these measures were designed to increase habitat diversity, the policy is considered to be too dilute to be effective (e.g. Pe'er *et al*. 2014). New AES under ‘Pillar 2’ are also about to be implemented, such as the English Countryside Stewardship Scheme. This scheme will be regionally targeted, competitive, and include packages of habitat options targeting pollinators and farmland birds (Natural England 2015). The packages are not compulsory, but applications are more likely to be successful if they meet the minimum requirements of a package.

Our results support evidence‐based packages of options in schemes (such as CG and the new Countryside Stewardship), and our findings suggest that these should improve habitat diversity and species richness beyond that of ELS. The success of the new Countryside Stewardship scheme will depend on the detail of the scheme design, along with the extent of uptake, monitoring, management resources and farmer training. The CG scheme offers an alternative funding model, which could increase the number of farms with packages of wildlife‐friendly farming options beyond that of Countryside Stewardship, given sufficient consumer demand and business subscription. We recommend that compulsory, contractually binding ecological standards should be part of future wildlife‐friendly farming schemes, in order to ensure efficient use of funding for biodiversity conservation in intensive agricultural landscapes.

## Supporting information


**Appendix S1**. Method details.
**Table S1.** Farm characteristics used in site selection.
**Table S2.** Results of Friedman chi‐square tests on habitat and landscape composition between scheme types.
**Table S3.** Farm habitat composition and 1‐km buffer landscape composition.
**Table S4.** Farm intensity parameters.
**Table S5.** List of local (100 and 250‐m radius) habitat categories in heterogeneity analysis.
**Table S6.** List of landscape (1 and 3‐km) habitat categories (adapted from the LCM 2007).
**Table S7.** General linear mixed effects model on habitat diversity as a function of scheme type and radius interaction (Gaussian errors).
**Table S8.** Results of GLMM models testing habitat diversity as a predictor of species richness.
**Table S9.** Most parsimonious models after simplification of GLMM models testing effects of scheme type and habitat diversity, plus their interaction on species richness.
**Fig. S1.** Scatter plots and regression lines for relationships between habitat diversity at the 100‐m radius scale and species richness of (a) plants, (b) butterflies, (c) solitary bees and (d) winter birds.
**Fig. S2.** Scatter plots and regression lines for relationships between habitat diversity at the 250‐m radius scale and species richness of (a) plants and (b) butterflies.
**Fig. S3.** Scatter plots and regression lines for relationships between habitat diversity at the 1‐km radius scale and species richness of solitary bees.
