# Supplementary material for: Supporting local diversity of habitats and species on farmland: a comparison of three wildlife‐friendly schemes
Source: J Appl Ecol. 2015 Nov 18;53(1):171–80. doi: 10.1111/1365-2664.12557 (PMC4982055; doi:10.1111/1365-2664.12557)
Supplement: Supplementary file 1 — Appendix S1. Method details. Table S1. Farm characteristics used in site selection. Table S2. Results of Friedman chi‐square tests on habitat and landscape composition between scheme types. Table S3. Farm habitat composition and 1‐km buffer landscape composition. Table S4. Farm intensity parameters. Table S5. List of local (100 and 250‐m radius) habitat categories in heterogeneity analysis. Table S6. List of landscape (1 and 3‐km) habitat categories (adapted from the LCM 2007). Table S7. General linear mixed effects model on habitat diversity as a function of scheme type and radius interaction (Gaussian errors). Table S8. Results of GLMM models testing habitat diversity as a predictor of species richness. Table S9. Most parsimonious models after simplification of GLMM models testing effects of scheme type and habitat diversity, plus their interaction on species richness. Fig. S1. Scatter plots and regression lines for relationships between habitat diversity at the 100‐m radius scale and species richness of (a) plants, (b) butterflies, (c) solitary bees and (d) winter birds. Fig. S2. Scatter plots and regression lines for relationships between habitat diversity at the 250‐m radius scale and species richness of (a) plants and (b) butterflies. Fig. S3. Scatter plots and regression lines for relationships between habitat diversity at the 1‐km radius scale and species richness of solitary bees. [file JPE-53-171-s001.pdf]

SUPPORTING INFORMATION

Appendix S1: Method details

HABITAT MAPPING

Habitat maps were ground-truthed using a handheld GPS enabled PC with Arc Pad software (accuracy ± 4m) using the habitat categories (Table S5). Hedgerows and tree lines were mapped using Google maps aerial images (Google 2013) and area was calculated by multiplying the length by a mean width of 1.93 m (data from 14 hedges in Berkshire and Oxfordshire, Garratt, M.P. pers. comm.). For buffers of 250 m and 100 m, roads, rivers and railways were digitised using a 1:25 000 scale OS map (minimum mappable unit of 0.01 ha).

WEIGHTED AREAS FOR PROPORTIONAL SAMPLING CALCULATIONS

Areas not in Environmental Stewardship (ES) were given a weighting of 1 and that were in ES were given a weighting calculated using the formula: Number of ES points / (85 x 0.9), which gives the lowest scoring ES option (EK2: Low input grassland: 85 points) a weighting of 1.05, and other areas in proportion to this, giving the highest scoring ES option (HE10 Floristically enhanced margin: 485 points) a weighting of 6.34.

HABITAT DIVERSITY CALCULATIONS

Areas less than 16m^2 within each buffer were removed from the calculations in light of the GPS accuracy (± 4m). The mean proportion of farm area that each buffer radius size included was: 100 m: 88 %, 250 m: 74 %, 1km: 40 %, 3km: 8 % (2012 random point data).

SCOPE OF SAMPLING

Summer biodiversity surveys were carried out between April and August, 2012-2014. Winter bird surveys were carried out on between January and March 2013. Insects were surveyed in 2012 and 2013 with three sampling rounds per year, summer birds were surveyed in 2013 and 2014 with five sampling rounds per year, winter birds were surveyed in 2013 over three sampling rounds and plants were surveyed in 2012 with one sampling round. In 2012 there were 10 insect and plant sampling stations per farm for the LW, CN and HD regions, and 15 per farm in the CS region. In 2013, there were 12 insect sampling stations per farm in all regions. Summer bird transects were divided into 10 sub-transects per farm and winter bird transects were divided into 30 sub-transects per farm. The total number of sampling stations across all years was 789.

Each insect sampling station consisted of a central point, a point 50 m north and one 50 m east, giving a 100 m insect transect and three pan trap points. For birds, line transects were chosen over point counts because they suit open, uniform habitats, low population densities and allow greater coverage (Gregory, Gibbons & Donald 2006). Summer bird transects were 2 km long, divided up proportionately using the methods described above, and placed along field boundaries due to restricted access to field centres. Winter bird transects were 3 km long, and the Chilterns North region was not surveyed. The winter transects were divided into 100-m long sub-transects, of which 2/3 were allocated to the perimeter of field boundaries and 1/3 were allocated to field centres. Mid-points of transects were taken as the centre of circular buffers used to calculate habitat diversity.

BUTTERFLY SAMPLING

Butterflies were recorded whilst walking the 100 m transects at a constant speed over a period of 10 minutes. Butterflies and bees observed within 2 m either side and in front of the observer were recorded to species level as far as possible. Wind speed was recorded using an anemometer, cloud cover using visual scale of 'oktas' and maximum temperature using a thermometer. As far as possible, the UK Butterfly Monitoring guidelines for weather conditions for transects were used, restricting survey occasions to when the temperature was over 13°C, between 0900 and 1700 hours.

BEE SAMPLING

Pan traps were made by painting plastic bowls with UV paint to form triplicate sets of one blue, one white and one yellow. Pan trapping is particularly good for sampling small solitary bees that are difficult to see with the naked eye and is less subjective than net sampling (Westphal *et al.* 2008). Contents of pan traps were collected after 24 hours. All three farms in a landscape were sampled as close together in time as possible, normally over a period of four days for logistical reasons. Each pan trap was half-filled with water to which a couple of drops of washing up liquid were added to reduce surface tension and facilitate the capture of insects.

BIRD SAMPLING

The standard Breeding Bird Survey (http:/www.bto.org/volunteer-surveys/bbs), methodology was adapted for this study. Summer surveys were carried out by local voluntary ornithologists, who surveyed the same farm all season. Winter surveys were carried out by Dominic Harrison. Surveyors walked at a steady speed of 25 m/min. Observations were assigned to categories based on the distance away from the transect line, and only observations within the distance bands 0-25 m and 25-100 m were included in analysis. Surveys were carried out between 6am and 9am, avoiding persistent heavy rain, poor visibility, and strong winds (exceeding Beaufort Force 4).

PLANT SAMPLING

Plants were surveyed in 1m^2 quadrats at each of the pan trap sampling points in 2012. All plants were recorded at 134 points.

References

Gregory, R.D., Gibbons, D.W. & Donald, P.F. (2006) Bird census and survey techniques. *Ecological survey techniques* (ed W.J. Sutherland), pp. 17–56. Cambridge University Press, Cambridge, UK.

Westphal, C., Bommarco, R., Carre, G., Lamborn, E., Morison, N., Petanidou, T., Potts, S.G., Roberts, S.P.M., Szentgyörgyi, H., Tscheulin, T., Vaissiere, B., Woyciechowski, M., Biesmeijer, J.C., Kunin, W.E., Settele, J. & Steffan-Dewenter, I. (2008) Measuring bee diversity in different European habitats and biogeographical regions. *Ecological Monographs*, **78**, 653–671.

Table S1: Farm characteristics used in site selection

Farm code	NCA	Soil type	Crops	Livestock	HLS (Y/N)	Farm size (ha)	Starting year
LW_ELS	Low Weald	Slowly permeable seasonally wet slightly acid but base-rich loamy and clayey soils	Wheat, barley, oats	Beef cattle, sheep	N	183.8	2007
LW_Org	Wealden Greensand	Slightly acid loamy and clayey soils with impeded drainage. Freely draining slightly acid loamy soils.	Barley-pea mix, turnips, lucerne, clover	Dairy cows	Y	344.5	1999
LW_CG	Low Weald	Slowly permeable seasonally wet slightly acid but base-rich loamy and clayey soils. Freely draining slightly acid loamy soils.	Wheat, OSR, oats, maize	Beef cattle, sheep	Y	344.7	2006
CS_ELS	Chilterns	Slightly acid loamy and clayey soils with impeded drainage. Freely draining slightly acid loamy soils.	Wheat, OSR, barley, maize, poppies	Beef cattle	N	295.4	2010
CS_Org	Chilterns	Slightly acid loamy and clayey soils with impeded drainage.	Wheat (ancient varieties)	Beef cattle, pigs	Y	144.5	1997
CS_CG	Chilterns	Slightly acid loamy and clayey soils with impeded drainage.	Wheat, OSR, barley, oats, linseed	Beef cattle	N	356.5	2004
CN_ELS	Chilterns	Slightly acid loamy and clayey soils with impeded drainage.	Wheat, oats, barley, OSR	Beef cattle, horses	N	475.7	2007
CN_Org	Chilterns	Slightly acid loamy and clayey soils with impeded drainage.	Wheat, oats, field beans, rye, spelt, clover	Beef cattle, horses	(Entered during study)	111.7	1998
CN_CG	Chilterns	Slightly acid loamy and clayey soils with impeded drainage. Freely draining lime-rich loamy soils.	Wheat, oats, barley, peas, field beans	Beef cattle, horses, sheep	Y	182.7	2006
HD_ELS	Hampshire Downs/Thames Basin Heaths	Shallow lime-rich soils over chalk or limestone. Slowly permeable seasonally wet slightly acid but base-rich loamy and clayey soils.	Wheat, barley, mustard, OSR	Sheep	N	672.1	2007
HD_Org	Hampshire Downs	Shallow lime-rich soils over chalk or limestone	Wheat, barley, oats, spelt, einkorn, clover	Sheep, cows, turkeys	Y	118.8	1999
HD_CG	Hampshire Downs	Shallow lime-rich soils over chalk or limestone. Freely draining slightly acid loamy soils	Wheat, barley, oats, OSR	Sheep	Y	266.8	2006

Table S2: Habitat composition by year for farms in the three schemes (proportion (%), mean ± SE over four farms per scheme type) and Friedman Chi^2 testing for differences between schemes

Year	Scheme type				
Habitat type	ELS	CG	Org	Friedman Chi^2 (2 df)	P value
**2012**					
AES grass	5.8 ± 3	15.2 ± 5.7	17.1 ± 6.7	0.13	0.94
AES margin	4 ± 1.3	9.4 ± 3.1	2 ± 1.2	3.5	0.17
Imp grass	20.4 ± 10	9.5 ± 4.4	22.4 ± 7.8	2	0.37
MFC	12.3 ± 7.3	22.5 ± 5.9	18.7 ± 7.3	1.5	0.47
Other	10.9 ± 6	9.5 ± 3.2	8.6 ± 4.8	1.7	0.42
Non-MFC	46.6 ± 10.7	33.9 ± 7.1	31.2 ± 12	2	0.37
**2013**					
AES grass	6 ± 3.1	14.5 ± 5.2	20 ± 6.1	2.8	0.25
AES margin	3.7 ± 1	7.6 ± 2.2	2 ± 1.3	3.5	0.17
Imp grass	20.5 ± 10.2	8.7 ± 3.8	25.9 ± 9.8	1.5	0.47
MFC	10.4 ± 6	7.2 ± 4.5	13.2 ± 4.5	0.6	0.75
Other	11.1 ± 6.2	10.2 ± 3.2	8.8 ± 4.9	0.5	0.78
Non-MFC	48.4 ± 13.9	51.8 ± 5	30 ± 16.6	2	0.37
**2014**					
AES grass	5.5 ± 2.9	13.8 ± 5	22.1 ± 3.9	5.7	0.06
AES margin	4.6 ± 2.2	8 ± 2.1	2.2 ± 1.3	3.5	0.17
Imp grass	22 ± 12.2	9.6 ± 4.7	21.8 ± 12.2	3.5	0.17
MFC	8.9 ± 8.9	6.9 ± 4.1	21.7 ± 8.5	0.9	0.63
Other	8.3 ± 3.8	12.3 ± 3.9	8.6 ± 4.8	1.7	0.42
**Hedgerow density**	40.5 ± 7.3	63.1 ± 8.1	72.5 ± 10.9	3.5	0.17

Table S3: Landscape composition differences between schemes (1km radius buffer around each farm in 2013, not including the farm, SNH = semi-natural habitat, MFC = mass flowering crop)

Region	Scheme	SNH (%)	MFC (%)	Organic (%)	(Sub)-urban (%)
Chilterns North	CG	8.13	5.45	0	6.02
	ELS	11.45	3.65	5.68	19.26
	Org	9.35	4.67	0	16.82
Chilterns South	CG	27.52	3.38	0.06	10.41
	ELS	19.85	0.75	0	13.07
	Org	23.57	0	0	16.5
Hampshire Downs	CG	5.54	0	0	0.85
	ELS	19.84	0	0.83	1.53
	Org	7.84	1.8	20.12	2.01
Low Weald	CG	22.62	0.82	0	1.15
	ELS	31.95	0.33	0.02	0.79
	Org	38.6	1.6	11.1	1.68
Friedman Chi^2 test (p value) on scheme differences (2 df)		1.5 (0.47)	2.5 (0.28)	1(0.61)	4.5(0.11)

Table S4: Farm intensity parameters

Region	Type	Mean no. of crops per year (±SE)	No. of insecticide products in 2012	Mean nitrogen fertiliser (N kg/ha) (±SE)	Mean wheat yield (t/ha) (±SE)	Stocking density in 2013 (LU/ha)	Mean field size (ha) (±SE)
Low Weald	ELS	2.67±0.33	6	149.91±12.7	6.85±0.23	0.33	4.48±0.46
	Organic	2.67±0.33	0	0	NA	0.86	7.50±0.92
	CG	2.33±0.33	4	225±41.3	8.56±0.49	0.62	7.31±0.92
Chilterns South	ELS	3.33±0.33	6	197.75±34.7	6.81±0.23	1.30	12.53±1.89
	Organic	1.00±0.00	0	0	2.29±0.36	0.48	4.28±0.37
	CG	5.33±0.33	6	168.4±23.8	7.72±0.42	1.22	11.32±1.20
Chilterns North	ELS	3.67±0.33	3	195±71.0	6.92±0.58	0.61	17.23±1.97
	Organic	6.67±0.88	0	0	2.99±0.19	1.31	7.90±1.05
	CG	2.00±0.58	2	159.07±25.2	NA	0.50	10.51±1.67
Hampshire Downs	ELS	2.00±0.58	3	180±20.8	7.48±0.40	0.37	11.93±1.34
	Organic	7.00±0.00	0	0	3.18±0.53	0.59	5.34±0.74
	CG	3.33±0.33	6	142.5±27.5	7.61±0.00	0.25	9.54±1.24

To check whether farms in different schemes varied in farm intensity, data on several intensity parameters was collected through farmer interviews (Table S4). Farmers were asked to provide data for the years 2011 – 2013, but the data farmers had available did not always cover all years. Differences between scheme types were tested using i) GLMMs with nested random effects for farm nested in region, for parameters data was available over several years or crop types, with scheme type differences tested using a likelihood ratio test, ii) Friedman Chi^2 for parameters where only one year of data was available and three scheme types were tested (n=12), iii) Welch's two-sample t-test for parameters where only one year of data was available and two scheme types were tested (n=8). The mean number of crops per year did not differ significantly between scheme types (Years: 2012-2014, GLMM, LRT, Chi^2= 1.42, df=2, n=36, p=0.491). The number of insecticide products used did not vary between CG and ELS farms (Year: 2012, t=0.392, df=5.48, n=8, p=0.710). The amount of synthetic nitrogen applied (kg/ha) did not differ significantly between CG and ELS farms (Years 2012 and 2013, GLMM, Likelihood ratio test, Chi^2 =0.079, df=1, n=30, p=0.779). The most frequently grown crop across all farms was wheat, so yield comparisons were only tested for wheat. Spring wheat was

grown by some organic farms and no non-organic farms. Overall, wheat yields differed significantly between schemes (Years: 2009-2013, GLMM, LRT, Chi^{2}= 13.70, df=2, n=52, p=0.001). Post-hoc tests revealed that wheat yields were significantly lower on organic farms compared to CG (p=0.001) and ELS (p=0.005). Stocking density did not differ significantly between scheme types (Year: 2013, Friedman Chi^{2} = 2, df = 2, n=12, p-value = 0.3679). Mean field size was significantly smaller on organic farms (Year: 2013, GLMM, LRT, Chi^{2}=5.43, df=2, n=327, n=12, p=0.066, post-hoc test: Org<ELS, p=0.021). Farm size did not differ significantly between scheme types (Year: 2013, Friedman Chi^{2}= 3.5, df = 2, n=12, p-value = 0.1738, Table S1).

Table S5: List of local (100-m and 250-m radius) habitat categories in heterogeneity analysis. Environmental Stewardship code descriptions available at www.naturalengland.org.uk, MFC = mass flowering crop, WBF = wild bird food, OSR=oilseed rape

2m buffer	Game cover	Lake	Scrub
6m buffer	Grass/clover grazed	Lucerne	Set aside
Arable bare	Grass/clover silage	Lucerne/Sanfoin silage	Winter spelt
Arable silage	Hay	Maize	Spring barley
Arable unknown	HC7	MFC	Spring beans
Barley	HD2	Mixed	Spring linseed
Barley+peas	HE10	Mustard	Spring oats
Barley+peas+grass	HE3	Neutral grassland	Spring wheat
Calcareous grassland	Heather and dwarf shrub	OE1	Stubble
Cereal	Heather grass	OE3	Suburban
Clover	Hedge	OF1	Track
Conifer	HF1	OF2	Tree planting
Deciduous	HF13	OG1	Two year fallow
Despoiled land	HF2NR	OK2	Urban
EE3	HF4	OK3	Urban industrial
EF1	HK10	One year fallow	WBF
EF2	HK15	Peas	Winter Barley
EF2NR	HK16	Pheasant pen	Winter beans
EF4	HK2	Pond	Winter oats
EF7	HK3	Poppy	Winter OSR
Einkorn	HK6	Railway	Winter rye
EK1	HK7	River	Winter spelt
EK2	HK8	Road	Winter wheat
EK2/Tree planting	HO1	Rough low-productivity grassland	
EK3	HO2	Saltmarsh	
Fallow	Improved		

Table S6: List of landscape (1-km and 3-km) habitat categories (adapted from the LCM 2007, Morton et al. 2011**)**

**BH SUB (adapted**)	
Acid grassland	Littoral sand
Arable bare	Maize
Arable unknown	MFC
Bare	Mixed
Calcareous grassland	Neutral grassland
Cereal	Polytunnel
Conifer	Pond
Deciduous	Recent woodland (<10 years)
Despoiled land	River
Fallow	Rough low-productivity grassland
Felled	Saltmarsh
Fen marsh and swamp	Scrub
Hay	Set aside
Heather and dwarf shrub	Suburban
Heather grass	Urban
Improved	Urban industrial
Lake	

Semi-natural habitats: Habitats included as semi-natural were selected using site-specific knowledge for their relative permanence compared to habitats created and managed through agri-environment schemes. On farm habitats included as semi-natural were species-rich grassland (HK15, HK6, HK7, HK8), chalk down (EK3, HK3), wet grassland (HK10), fen and marsh (EK3), heathland (HO1, HO2), woodland (including HC7) and scrub. Land use types included were Deciduous, Conifer, Fen marsh and swamp, Heather and dwarf shrub, Heather grass, Acid grassland, Calcareous grassland, Neutral grassland, Rough low-productivity grassland, Saltmarsh, Scrub, Mixed woodland.

Table S7: General linear mixed effects model on habitat diversity as a function of scheme type and radius interaction (Gaussian errors)

Marginal R^2: 0.461, Conditional R^2:0.513

Random effects:

Random effects:					
Groups	Name		Variance	Std.Dev.	
Point:(Farm:(Region:Year))	(Intercept)		0.012755	0.11294	
Farm:(Region:Year)	(Intercept)		0.004187	0.06471	
Region:Year	(Intercept)		0.015679	0.12522	
Year	(Intercept)		0	0	
Residual			0.089028	0.29838	
Fixed effects:					
	Estimate	Std. Error	df	t value	Pr(>|t|)
(Intercept)	0.69696	0.04923	29.7	14.157	<0.001
TypeCG	0.17714	0.04726	80	3.748	<0.001
TypeOrg	0.119	0.04726	80	2.518	0.0138
Radius0.25	0.53935	0.03664	1209.9	14.722	<0.001
Radius1	0.95719	0.03659	1196.3	26.16	<0.001
Radius3	0.95189	0.03659	1196.3	26.016	<0.001
TypeCG:Radius0.25	0.05736	0.05178	1203.1	1.108	0.2681
TypeOrg:Radius0.25	0.12562	0.05178	1203.1	2.426	0.0154
TypeCG:Radius1	-0.08609	0.05175	1196.3	-1.664	0.0964
TypeOrg:Radius1	-0.03444	0.05175	1196.3	-0.666	0.5058
TypeCG:Radius3	-0.22112	0.05175	1196.3	-4.273	<0.001
TypeOrg:Radius3	-0.0442	0.05175	1196.3	-0.854	0.3931

Table S8: Results of GLMM models testing habitat diversity as a predictor of species richness.

	Radius (km)	Estimate	Std Error	LRT Chi^2	p value	Marginal R^2	Conditional R^2
**Plants**							
	**0.10**	**0.470**	**0.150**	**12.13**	**<0.001**	**0.500**	**0.558**
	**0.25**	**0.418**	**0.164**	**6.291**	**0.012**	**0.440**	**0.491**
	1.00	-0.003	0.355	0.048	0.826	<0.001	0.009
	3.00	0.138	0.464	0.088	0.766	0.659	0.691
**Butterflies**							
	**0.10**	**0.535**	**0.099**	**30.71**	**<0.001**	**0.939**	**0.940**
	**0.25**	**0.330**	**0.121**	**7.44**	**0.006**	**0.940**	**0.940**
	1.00	0.338	0.208	2.56	0.110	0.949	0.949
	3.00	0.344	0.210	2.56	0.110	0.948	0.948
**Bumblebees**							
	0.10	-0.090	0.093	0.949	0.330	0.723	0.723
	0.25	-0.158	0.110	2.043	0.153	0.716	0.716
	1.00	-0.117	0.190	0.376	0.540	0.703	0.703
	3.00	-0.213	0.206	1.084	0.298	0.697	0.697
**Solitary bees**							
	**0.10**	**0.197**	**0.078**	**6.055**	**0.014**	**0.286**	**0.334**
	0.25	0.137	0.101	1.771	0.183	0.310	0.355
	**1.00**	**-0.463**	**0.179**	**4.743**	**0.029**	**0.621**	**0.628**
	3.00	-0.422	0.001	2.330	0.127	0.585	0.596
**Birds (summer)**							
	0.10	0.003	0.043	0.004	0.948	0.999	0.999
	0.25	-0.023	0.042	0.303	0.582	0.999	0.999
	1.00	0.122	0.088	0.121	0.088	0.999	0.999
	3.00	0.023	0.131	0.031	0.861	0.999	0.999
**Birds (winter)**							
	**0.10**	**0.178**	**0.069**	**6.314**	**0.012**	0.144	0.145
	0.25	0.054	0.078	0.456	0.498	0.195	0.198
	1.00	-0.281	0.172	2.686	0.101	0.572	0.575
	3.00	0.011	0.118	0.009	0.926	0.240	0.245

Table S9: Most parsimonious models after simplification of GLMM models testing effects of scheme type and habitat diversity, plus their interaction on species richness

Taxonomic group	Scale	Variable	Estimate	SE	df	LRT Chi^2	P value
Plants							
	100 m						
		Scheme type			2	7.78	0.020
		CG	-0.0008	0.201			
		Org	0.0022	0.194			
		Habitat diversity	-0.0003	0.130	1	13.2	<0.001
	250 m						
		Scheme type			2	6.32	0.043
		CG	-0.015	0.202			
		Org	0.468	0.194			
		Habitat diversity	0.407	0.165	1	5.93	0.015
Butterflies							
	100 m						
		Abundance	5.889	0.526	1	96.77	<0.001
		Habitat diversity	0.530	0.098	1	29.87	<0.001
		Scheme type			2	6.25	0.044
		CG	0.186	0.122			
		Org	0.286	0.114			
		MFC 1km	0.013	0.005	1	8.21	0.004
		Year	0.540	0.105	1	27.63	<0.001
	250 m						
		Abundance	6.04	0.520	1	106.00	<0.001
		Habitat diversity	0.293	0.124	1	5.60	0.018
		Scheme type			2	5.26	0.072
		CG	0.216	0.123			
		Org	0.248	0.114			
		MFC 1km	0.012	0.005	1	6.93	0.008
		Year	0.556	0.105	1	29.39	<0.001

Taxonomic group	Scale	Variable	Estimate	SE	df	LRT Chi^2	P value
Bumblebees							
	100 m						
		Abundance	1.587	0.217	1	41.7	<0.001
		Habitat diversity	-0.096	0.093	1	1.07	0.300
		Scheme type			2	1.70	0.427
		CG	0.029	0.108			
		Org	-0.106	0.111			
		MFC 1km	0.007	0.004	1	2.41	0.121
		Year	1.668	0.133	1	216	<0.001
	250 m						
		Abundance	1.599	0.214	1	42.8	<0.001
		Habitat diversity	-0.157	0.114	1	1.89	0.169
		Scheme type			2	1.42	0.491
		CG	0.047	0.110			
		Org	-0.080	0.114			
		MFC 1km	0.008	0.004	1	3.08	0.08
		Year	1.679	0.133	1	217	<0.001
Solitary bees							
	100 m						
		Abundance	1.118	0.125	1	74.52	<0.001
		Habitat diversity	0.196	0.079	1	6.007	0.014
		Scheme type			2	1.155	0.561
		CG	-0.027	0.128			
		Org	-0.138	0.129			
		MFC 1km			1	7.393	0.007
		Year			1	35.229	<0.001
	250 m						
		Abundance	1.166	0.125	1	80.70	<0.001
		Habitat diversity	0.144	0.102	1	1.948	0.163
		Scheme type			2	1.378	0.502
		CG	-0.023	0.132			
		Org	-0.152	0.132			
		MFC 1km	0.012	0.005	1	6.415	0.011
		Year	0.456	0.076	1	33.988	<0.001

Taxonomic group	Scale	Variable	Estimate	SE	df	LRT Chi^2	P value
Summer birds							
	100 m						
		Abundance	0.307	0.030	1	88.07	<0.001
		Habitat diversity				0.003	0.959
		Scheme type			2	1.117	0.572
		CG	-0.029	0.049			
		Org	-0.057	0.055			
		Year	0.172	0.039	1	18.35	<0.001
	250 m						
		Abundance	0.307	0.030	1	18.297	<0.001
		Habitat diversity	-0.015	0.043	1	0.118	0.732
		Scheme type			2	0.933	0.673
		CG	-0.027	0.048			
		Org	-0.053	0.056			
		Year	0.175	0.040	1	18.297	<0.001
Winter birds							
	100 m						
		Abundance	0.102	0.005	1	382.5	<0.001
		Habitat diversity	0.192	0.068	1	7.46	0.006
		Scheme type			2	2.36	0.307
		CG	-0.024	0.064			
		Org	0.073	0.066			
	250 m						
		Abundance	0.107	0.005	1	490.2	<0.001
		Habitat diversity	0.073	0.081	1	0.80	0.372
		Scheme type			2	1.56	0.459
		CG	-0.044	0.065			
		Org	0.042	0.067			

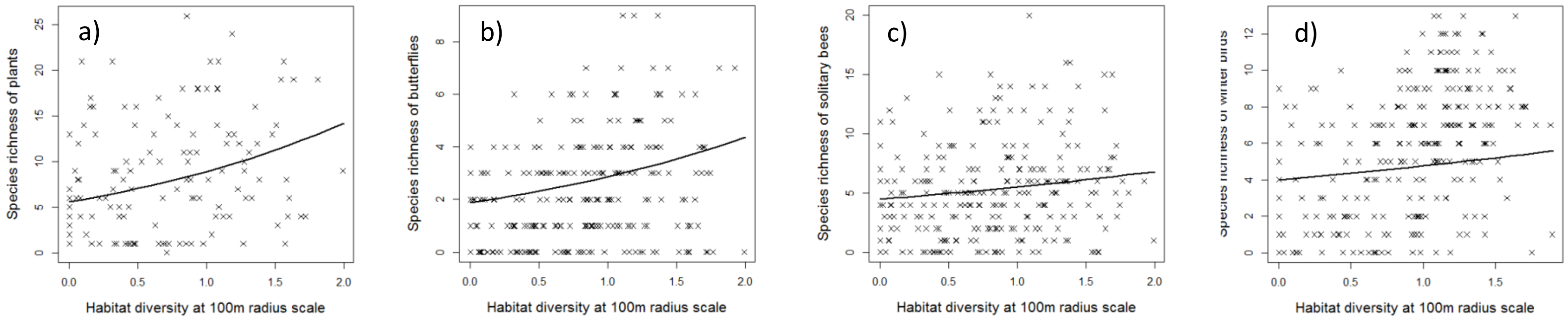

Figure S1: Scatter plots and regression lines for relationships between habitat diversity at the 100 m radius scale and species richness of a) plants, b) butterflies, c) solitary bees and d) winter birds

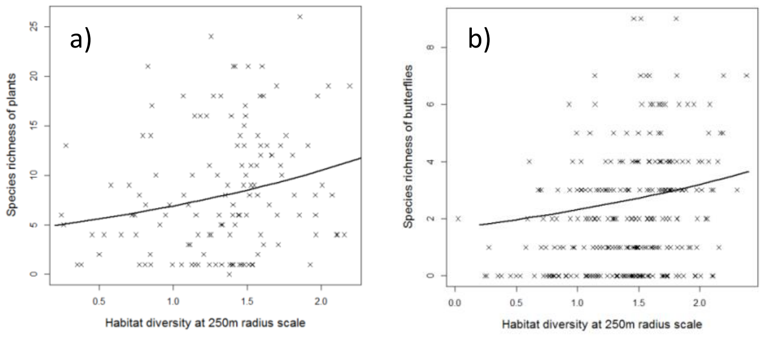

Figure S2: Scatter plots and regression lines for relationships between habitat diversity at the 250 m radius scale and species richness of a) plants and b) butterflies.

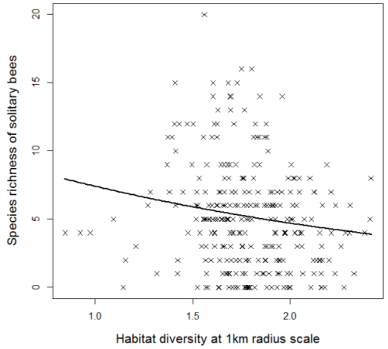

Figure S3: Scatter plots and regression lines for relationships between habitat diversity at the 1 km radius scale and species richness of solitary bees.
